# Oropouche virus detection in saliva and urine

**DOI:** 10.1590/0074-02760190338

**Published:** 2020-02-27

**Authors:** Valdinete Alves do Nascimento, João Hugo Abdalla Santos, Dana Cristina da Silva Monteiro, Karina Pinheiro Pessoa, Antonio José Leão Cardoso, Victor Costa de Souza, Ligia Fernandes Abdalla, Felipe Gomes Naveca

**Affiliations:** 1 Fundação Oswaldo Cruz-Fiocruz, Instituto Leônidas e Maria Deane, Manaus, AM, Brasil; 2 Fundação Oswaldo Cruz-Fiocruz, Instituto Oswaldo Cruz, Programa de Pós-Graduação em Biologia Celular e Molecular, Rio de Janeiro, RJ, Brasil; 3 Universidade Federal do Amazonas, Manaus, AM, Brasil; 4 Hospital Adventista de Manaus, Manaus, AM, Brasil; 5 Fundação Oswaldo Cruz-Fiocruz, Instituto Leônidas e Maria Deane, Programa de Pós-Graduação em Biologia da Interação Patógeno-Hospedeiro, Manaus, AM, Brasil; 6 Universidade do Estado do Amazonas, Manaus, AM, Brasil

**Keywords:** arboviruses, orthobunyavirus, Oropouche virus, saliva, urine, real-time polymerase chain reaction

## Abstract

Oropouche virus (OROV) is an arthropod-borne virus of the *Peribunyaviridae* family, transmitted to humans primarily by *Culicoides paraensis*. It is one of the main arboviruses infecting humans in Brazil, primarily in the Amazon Region. Here, we report the detection of OROV in the saliva and urine of a patient whose samples were collected five days after the onset of symptoms. Nucleotide sequencing and phylogenetic analysis further confirmed the results. To our knowledge, this is the first study reporting the detection of OROV in the saliva and urine of an infected patient. In addition, the results of our study expand the current knowledge pertaining to the natural history of Oropouche fever.

The Oropouche virus (OROV) is an arthropod-borne virus, with a triple-segmented negative-stranded linear RNA genome. Each segment is designated according to its size as L (large), M (medium), and S (small). This arbovirus belongs to the *Peribunyaviridae* family, genus *Orthobunyavirus*, species *Oropouche orthobunyavirus* (https://talk.ictvonline.org/taxonomy/), and two invertebrate vectors have been associated with its urban transmission cycle, namely, *Culicoides paraensis* (Ceratopogonidae), which is considered the primary vector, and *Culex quinquefasciatus* (Culicidae).^1^ Recently, one study reinforced the potential role of *Culex sp.* mosquitoes in OROV transmission.^2^

An infection with OROV can result in an acute febrile and exanthematous illness, with symptoms frequently similar to other viral infections such as dengue. Oropouche fever cases were reported in several Brazilian states, including Amazonas, Acre, Bahia, Pará and Mato Grosso, as well as in other South American countries.^2^ ^,^ ^3^ ^,^ ^4^ ^,^ ^5^ ^,^ ^6^ ^,^ ^7^ ^,^ ^8^ ^,^ ^9^ ^,^ ^10^

Oropouche fever is usually confirmed by detecting the OROV genome in the plasma or sera of acutely infected patients, or by specific IgM serology during convalescence.^1^ ^,^ ^11^ ^,^ ^12^ Nevertheless, recent studies have shown arbovirus detection using other body fluids, such as saliva and urine. This was demonstrated for different viral species such as Chikungunya virus (CHIKV, family *Togaviridae*, genus *Alphavirus*),^13^ ^,^ ^14^ as well as Dengue virus (DENV),^15^ West Nile virus (WNV),^16^ and Zika virus (ZIKV),^17^ ^,^ ^18^ ^,^ ^19^ which are members of the family *Flaviviridae*, genus *Flavivirus*. That said, the orthobunyavirus genus has not been detected in saliva or urine, to date. Therefore, this study aimed to investigate the presence of OROV in these biological specimens, during the acute phase of the illness.

Between February and June 2016, the period at the beginning of the ZIKV epidemic in the Amazonas State, patients who visited the Hospital Adventista de Manaus presenting symptoms suggestive of an arbovirus infection were invited to participate in the present study. A total of 352 acute-phase specimens, collected amid 0 (first 24 h) to five days after onset of symptoms, were sent to Instituto Leônidas e Maria Deane - Fiocruz (ILMD), a research unit of the Brazilian Ministry of Health that was responsible for the laboratory diagnosis of ZIKV during its emergence in the Amazonas State, Brazil. Plasma samples were subjected to RNA extraction using the QIAamp Viral RNA Mini Kit (Qiagen), according to the manufacturer’s instructions. Subsequently, we tested all samples for ZIKV,^20^ CHIKV,^21^ and DENV,^22^ by reverse transcription quantitative real-time polymerase chain reaction (RT-qPCR). A multiplex RT-qPCR assay further tested negative samples for Mayaro virus (MAYV) and OROV.^11^

We evaluated the saliva and urine from five OROV-positive patients (from plasma analyses), as well as 50 other randomly chosen patients whose plasma samples were negative for all arboviruses tested, using the same protocol. Subsequently, the OROV-positive samples were subjected to conventional RT-PCR, targeting a fragment of the L, M, and S segments using a protocol developed during this study. Initially, we performed the reverse transcription reaction using SuperScript IV Reverse Transcriptase and random primers (Thermo Fisher Scientific). The cDNA was PCR amplified in a reaction using 1.5 mM Mg^2+^, 0.2 mM of dNTPs, 1 U of Platinum Taq DNA Polymerase (Thermo Fisher Scientific) and 0.3 µM of specific primers for S and L segments, and 0.5 µM for the M segment (Table).

**TABLE:** Oligonucleotides designed and used in this study

Oligo	Sequence (5’- 3’)	Start	Stop
OROV_L_56_FNF	TTGCTCAACCARTATCGRAATAGGAT	56	81
OROV_L_174_FNF	CTGCAAAYCTTGAGTAYAGAAATGATG	174	200
OROV_L_621_FNR	TCAATCCATGGCAATGTCATTGT	621	599
OROV_M_2185_FNF	TCCCAAATCTAATCCTTTTACYGAT	2185	2209
OROV_M_2864_FNF	AGTATAGATGTACAAGGTACAGAATC	2864	2889
OROV_M_3564_FNR	TTCCTTCTCATAGCATGGCAT	3564	3544
OROV_S_6_FNF	TGTACTCCACAATTCAAAACAT	6	27
OROV_S_133_FNF	ACGGACAAGTGCTCAATGCT	133	152
OROV_S_728_FNR	TCCGAATTGGCGCAAGAAGT	728	709
Assay	Primer pairs		Size (bp)
L - 1^st^ PCR (55ºC)	OROV_L_56_FNF + OROV_L_621_FNR		566
L - semi-nested (50ºC)	OROV_L_174_FNF + OROV_L_621_FNR		448
M - 1^st^ PCR (55ºC)	OROV_M_2185_FNF + OROV_M_3564_FNR		1380
M - semi-nested (55ºC)	OROV_M_2864_FNF + OROV_M_3564_FNR		701
S - 1^st^ PCR (58ºC)	OROV_S_6_FNF + OROV_S_728_FNR		723
S - semi-nested (58ºC)	OROV_S_133_FNF + OROV_S_728_FNR		596

Start/Stop positions refers to the nucleotide position of OROV GenBank reference sequence NC_005776.1 (segment L), NC_005775.1 (segment M), and NC_005777.1 (segment S). To increase sensitivity, we developed semi-nested reactions for each genome segment. All 1st polymerase chain reaction (PCR) reactions followed the same program: 94ºC for 2 min for enzyme activation; 35 cycles (94ºC for 30 s, 55 or 58ºC for 30 s, and 72ºC during 1 min/Kb), a final step at 72ºC for 5 min. For semi-nested reactions: 94ºC for 2 min for enzyme activation; 30 cycles (94ºC for 30 s, 50, 55 or 58ºC for 30 s, and 72ºC for 1 min), a final step at 72ºC for 5 min. All primers used in this study were synthesised by IDT DNA Technology, USA.

The nucleotide sequencing reaction was carried out on an ABI3130 Genetic Analyzer at the ILMD genomics platform. The data were analysed using the Geneious software v10.2.6^23^ for quality check, trimming, and contig assembly. The genome segments sequenced in this study were analysed together with three different datasets, one for each segment, containing 75 species of orthobunyavirus, recognised by the International Committee on Taxonomy of Viruses (ICTV - Virus Metadata Repository: version June 1, 2019; MSL34 - https://talk.ictvonline.org/taxonomy/vmr/m/vmr-file-repository/8287/download), with the full genome records available in GenBank on 01-Jun-2019. All sequences in the datasets were aligned with the partial sequences of each genome segment generated in this study using MUSCLE (codons), embedded in the MEGA X software.^24^ Species confirmation was performed using phylogenetic reconstruction by Bayesian Inference (BI) with MrBayes 3.2.6 with two runs and 20 million Markov chain Monte Carlo (MCMC) generations^25^ at CIPRES Science Gateway V. 3.3 (https://www.phylo.org) and maximum-likelihood (ML) with PhyML 3.0^26^ with Smart Model Selection (SMS)^27^ (http://www.atgc-montpellier.fr/phyml/). All procedures in this study were in accordance with guidelines of the Ethics Committee of the State University of Amazonas (CAAE: 56.745.116.6.0000.5016).

Among the tested plasma samples, 202 were positive for ZIKV, one for CHIKV, three for DENV, and five for OROV. As previously described in this manuscript, all OROV-positive patients had their saliva and urine further evaluated. A 51-year-old female patient (BR_AM_ILMD_0240AOS_2016), living in Manaus, Amazonas State, Brazil, whose samples were collected on 2016-04-11, five days after the onset of symptoms, was positive for OROV in both saliva and urine, with Ct values of 31 and 26, respectively. According to her medical records, she presented with a fever, rash, myalgia, pruritus, headache, arthralgia, lymphadenopathy, diarrhea, and vomit during her illness. All of the other 50 saliva and urine specimens tested from patients with no arboviral infection remained negative for OROV.

Partial coding sequence (CDS) sequencing was successful for the L (396 bp), M (648 bp), and S (555 bp) segments and these sequences were used for phylogenetic reconstruction, using a dataset of ICTV recognised orthobunyavirus species. Both BI and ML phylogeny were evaluated using the nucleotide substitution model GTR+G+I, as selected by the SMS approach. All Bayesian runs reached convergence with an average standard deviation of split frequencies lower than 0.009 and ESS values > 200. For the three genome segments, the sample BR_AM_ILMD_0240AOS_2016 clustered with the OROV RefSeq with high (1.0) posterior probability support (Figure). The same topology, with high support, was observed in the ML tree (data not shown).

**Figure:**
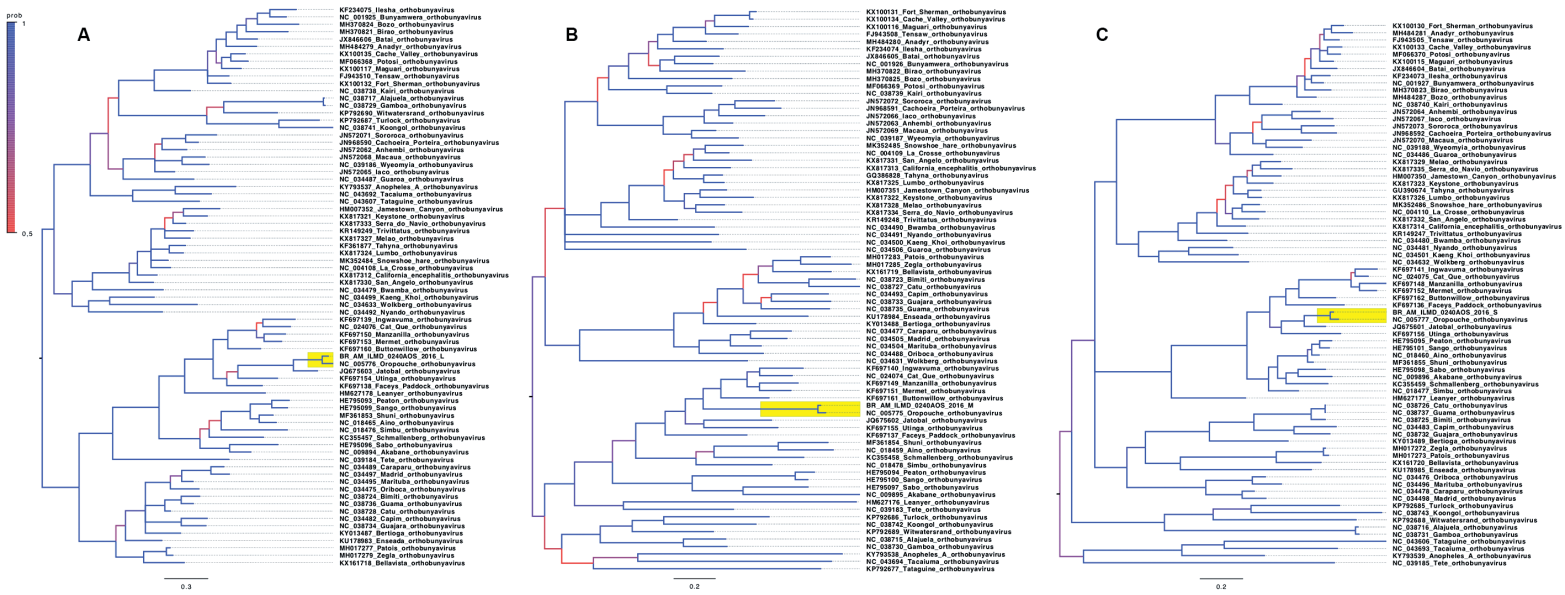
Phylogenetic tree of Orthobunyavirus species. Three Bayesian trees, one for each genome segment, were constructed with MrBayes software v3.2.6 and 76 taxa (the 75 orthobunyavirus species recognised by the International Committee on Taxonomy of Viruses (ICTV) with complete genome records available in GenBank on 01-Jun-2019 and the sample BR_AM_ILMD_0240AOS_2016 reported in this study). Phylogenetic trees were set mid-rooted, with increased node order in FigTree 1.4.4 for clarity. A colour-key represents the posterior probability values of each branch. The clade containing the sequence described in this study is highlighted in yellow, clustered with the Oropouche virus RefSeq. The scale bar represents nucleotide substitutions per site. A: L segment tree; B: M segment tree; C: S segment tree.

Several reports have shown that different arboviruses like ZIKV and CHIKV can be detected by testing unusual body fluids, such as saliva and urine. Two studies with samples collected from patients infected with ZIKV showed that some individuals were positive only in saliva and not in serum.^17^ ^,^ ^28^ Interestingly, our group found similar results during the emergence of ZIKV in the Amazonas State, Brazil (unpublished observations). Other reports show that saliva may serve as an alternative specimen for CHIKV detection during the acute phase of illness, with positivity ranging from 58.3-77%.^13^ ^,^ ^14^ However, no previous study has reported the detection of a member of the *Orthobunyavirus* genus in these biological fluids.

Therefore, we decided to investigate if OROV, an endemic arbovirus in the Amazon region, could also be identified using the same biological specimens. In the present study, OROV was detected by RT-qPCR in the saliva and urine of a patient, whose specimens were collected five days after the onset of symptoms. This result was further confirmed by conventional RT-PCR, followed by nucleotide sequencing and phylogenetic analysis using the ICTV reference database for orthobunyaviruses, which clustered the sequences of all the three partial genomic segments obtained in this work, with the OROV RefSeqs.

It was beyond the scope of the present study to assess the best human specimen for OROV detection; interestingly though, we found a higher viral load in urine, as suggested by the lower Ct value observed in this specimen, compared to the results for both, saliva and plasma. Since we evaluated OROV-positivity in the urine and saliva samples of only one patient, further studies are necessary to better evaluate the viral loads in these specimens. Furthermore, future studies should also comprehensively evaluate these body fluids for infectious OROV particles. Previous studies have also reported the detection of arboviruses in urine. One study with WNV, an arbovirus of the *Flaviviridae* family, reported a higher viral load in urine than in plasma during the acute phase of the illness.^16^ However, two different studies with CHIKV and DENV showed a significantly lower rate of detection when urine was tested during the first few days after the onset of symptoms, as compared to samples collected during the second week of illness.^14^ ^,^ ^15^ Together, these results suggest that urine may be used as a specimen for the detection of different arboviruses. However, longitudinal studies, with a more significant number of patients, need to be carried out to evaluate the potential use of different body fluids for OROV detection.

To our knowledge, this is the first study reporting the detection of OROV in the saliva and urine of an infected patient, suggesting that these specimens should be further evaluated as alternative sources for the detection of OROV. Furthermore, this result also raises the question of whether other members of the *Peribunyaviridae* family can be detected in a similar manner. Finally, the detection of OROV in urine and saliva strongly suggests that this virus sheds into additional body fluids other than blood and the cerebrospinal fluid, as previously reported.^29^ Therefore, our results may further contribute to the current knowledge pertaining to the natural history of Oropouche fever.

*Nucleotide sequence accession number* - The partial sequences of the OROV isolate BR_AM_ILMD_0240AOS_2016 are available in GenBank, under the accession numbers MN419356 (L), MN419357 (M), and MN419358 (S).
